# Miscellanies

**Published:** 1834-01-01

**Authors:** 


### Miscellanies.

### Mr. Guthrie's Ligature of the common Iliac Artery.

The lady, whose common iliac artery Mr. Guthrie tied on the right side, on the 24th of August last, for a large gluteal aneurism, left town on Saturday, the 21st inst., for Scotland, in good health and spirits. The tumour has diminished three-fourths in size—the pain she experienced in the foot and leg, from the pressure of the aneurism on the great sciatic nerve, has nearly subsided—the pulsation of the femoral artery, immediately below Poupart's ligament is as distinct and full as that on the opposite side. Thus this most formidable operation has been successfully performed for the first time; and while it adds a wreath of laurel to the brows of the distinguished surgeon, it exhibits a splendid triumph of British surgery.

### Medical Reform.

The weekly journals will have informed our brethren in all parts of the Empire, and even beyond the seas, that the struggle has commenced between the profession at large, and the corporate bodies by which they are oppressed—or, at all events, unaided in any useful matter of medical legislation. The struggle will be great—perhaps protracted. Unless every large town in the kingdom petitions Parliament promptly, we shall have no reform that is worth a straw. It is probable that, at this moment, the dye is cast in favour of a royal commission, instead of a parliamentary enquiry! The profession can only work by open and fair petition—the corporate bodies are all at work—in the sick-rooms, the nurseries—nay, in the water-closets, to foil every attempt at reform! Every stratagem, every ruse, every species of *Punic* promise, will be put in force to keep up corporate monopoly and antiquated privileges. The apple of discord will be launched from a hundred hands, and in a hundred directions, every hour in the day. Some of our contemporaries are too sure of medical reform, and their confident prognostications will tend to lull the profession, especially in the provinces, into a fatal security. As for ourselves, we candidly confess that we are any thing but sanguine of much amelioration for years to come. There is such a powerful phalanx under the influence of the corporate bodies, that reform will have a desperate fight and a doubtful victory. One of the greatest evils in the profession—the union of pharmacy with medical practice—will be fostered, not only by the corporate bodies, but by nine-tenths of the two great ORDERS of the profession. The Apothecaries' Company well know, that were the general practitioners emancipated from the thraldom of *trade*, and could dedicate themselves to the *science* of their profession, the expenditure of drugs would, at once, be *diminished to one-fourth of its present rate.* As a trading company, the Hong-merchants of Blackfriars are well aware of this contingency, and will oppose every parliamentary measure that has a tendency to dissociate pharmacy from the practice of medicine. The other corporate bodies will pull the same way, for obvious reasons—a jealousy of the encroachment of the "TIERS ETAT" on the NOBLESSE of the profession. The example of the French, who are about to dissolve effectually the unhallowed tie that binds physic and pharmacy together, will be some advantage to us here, though this same example will be eyed by the anti-reformers with as much detestation and terror, as constitutional liberty is viewed in Vienna, Berlin, and St. Petersburgh. The general practitioners, then, should not hope too much from Parliament in redress of this paramount evil. They ought to undertake the cure themselves. The remedy may be somewhat difficult; but we are confident that it is in their own hands. It will require unanimity and extensive combination. This combination, fortunately is of a very different kind from that of the trades' unions. The most aristocratic sticklers for their different ORDERS deplore, daily and hourly, the baneful system of remunerating the skill of the practitioner by one thousand per cent. profit on salts and senna!! The practitioner feels the degradation of such a system, and the patient feels it—if not in mind, at all events in body—and nine-tenths of the community would gladly see the mode of remuneration changed. It is, therefore, for the general practitioners to set about this item of reform as soon as possible, without waiting for the doubtful chance of legislative enactments.

We hope, however, to see the day when the level of medical education shall be raised so high as to separate physic from pharmacy completely. It is absurd to say that we must have poor men's doctors, the same as poor men's plasters—one scale of education for the rich, and another for the poor. What are our hospitals and dispensaries for? What are the morning levees of junior practitioners for, where the poor are treated gratuitously? There is a great redundancy of medical practitioners in this country, and what remedy can possibly be less objectionable than that of raising the education of the general practitioner up to the level at which the PURES now stand. There will still be plenty of operatives in the market—more than will supply the demand—although the article offered for sale will be much improved in quality. The dissociation of physic from pharmacy would be immediately productive of one immense advantage, the emancipation from apprenticeship. The Pures, both in physic and surgery, cannot pretend that an apprenticeship is necessary for the practice of either or both of these branches, since they themselves do not undergo that degrading yoke. The moment, therefore, that the general practitioner discards the sale of drugs, and charges for his professional skill, apprenticeship will only appertain to the chemist and druggist.

One of our contemporaries is quite shocked at the idea of raising the education of the general practitioner to a level with that of the Scotch or Irish diplomatist. "Will the worthy Doctor (J. Johnson) allow us to ask him one question? Does he mean to say that the candidate practitioner or candidate member of the faculty will be required to possess an amount of education equal to that taught by the Irish and Scotch universities?"

We sincerely thank our contemporary for this interrogatory. We unhesitatingly answer in the affirmative. We aver that the education of the general practitioner is now very nearly as good, and *ought be* somewhat better, than that of the Scotch Doctor who asks the question. We appeal to every one, including our contemporary himself, whether the examination of the general practitioner is not now more searching than that of the physician in Pall Mall East, the surgeon in Lincoln's-Inn Fields, or the graduate in Edinburgh and Glasgow? And yet our contemporary entertains as great a horror of an equilibrium in education, as Nature does of a vacuum in the atmosphere! Is it not preposterous that the examination of the general practitioner should be more severe than that of the *ranks* above-mentioned, while their preliminary education should be less? Let our contemporary answer that question in return.

"When, says he, the clamour ceases against the Society of Apothecaries for requiring *so much* in their curriculum, we shall believe Dr. Johnson answering in the affirmative—but not till then." Now let us analyse this jesuitical declaration of our contemporary. Who has raised a clamour against the curriculum of the Society, as far as the essential studies of medical science are concerned? None. But we have clamoured, and shall continue to clamour, so long as we have tongues to speak or pens to write, against the barbarous part of the curriculum, that consigns a youth to five years' labour *in the shop*, rolling pills and filtering tinctures, while only two years are assigned by the sapient society to the acquirement of anatomy, physiology, surgery, chemistry, therapeutics, and clinical instruction! Our contemporary is horrified at the idea of general practitioners rising to a par with the Scotch doctor, and very wisely advocates the system of keeping him back in the ranks of science by a five years' apprenticeship behind the counter.* Let the Apothecaries' Company reverse the order of their curriculum, and make the apprenticeship two years, while the study of the other branches is to occupy five years and then we shall approve of it. *Then* the general practitioner will be far superior to the Scotch doctor, and that "*Utopian democracy of medicine,*" which our contemporary so justly dreads, will be in a fair way for realization. The curriculum of the Apothecaries is unjust. The Society expects the candidate to undergo a rigid scrutiny in the various branches of medical science, and yet annihilates five years out of the seven dedicated to the acquisition of that science!

To the delicate ears of the mouth-piece of the medical aristocracy, the word "REPUBLIC" must sound harsh and discordant as the hyena's howl. Yet the term has been applied to LETTERS, of which medicine forms no inconsiderable department. Not being under the trammels of a faction—we are free to confess that we do anticipate a republic of medical science and literature, as well as of letters and science in general—and that a day will come when *rank*, in medicine, will depend on individual talent and acquirement—not on the name of the place where the science of medicine was acquired. That day is fast approaching, notwithstanding the ridicule which our contemporary attempts to throw upon it. We strongly suspect that he feels a conviction of it himself:—and if he does not, he is either obtuse in his intellect, or he mingles little with the various *ranks* of his brethren. When he mixes more with them, he will be inclined to stickle

---

* The question which our contemporary has put to Dr. Johnson, respecting the high scale of education possessed by the Scotch physician, comes with a very bad grace from a journalist, who has uniformly maintained that the Scotch physician ought to undergo an examination by the Society of Apothecaries, before he is allowed to act as a general practitioner in England! This is certainly an Irish kind of proof, that the said Scotch Doctor possesses a higher grade of education and knowledge than the general practitioners of this country!

less for "his ORDER," and to entertain a faint idea that the *capacity* of the "democracy of medicine" is just as good as that of the aristocracy—and that every attempt to prevent uniformity, or even equality of *education*, will, in the end, be fruitless. We are not so Utopian as to suppose that equalization of education will produce equalization of talent, or of knowledge. Far from it. If every individual in the profession were to undergo the same course of study, and the same kind of examination, there would be just as much inequality of *real rank* as at present. But it would be founded on a very different basis—not on the name of the river or frith where the knowledge was acquired, but on the quantum which the individual possessed, and his talent for using it.

Our contemporary ridicules the idea of one faculty for the regulation of medical education and practice as perfectly Utopian. Now of the three Corporate Bodies in London, ONE, and that the lowest in rank, regulates the education and practice of nine-tenths of the medical profession in England and Wales. It would not require a very great stretch of imagination to suppose that it might regulate the remaining tenth also. For our own parts, we see no reason why any one of the three Corporate Bodies might not be erected into a faculty for presiding over the whole. The College of Surgeons have no legal power at all—and the College of Physicians have none, except over their own body in London and seven miles around. Can there be any very insurmountable difficulty in forming a FACULTY for superintending the education and practice of the profession, elected out of the three Corporate Bodies now existing? The various colleges in the kingdom may remain as seminaries of education, with the power of granting degrees, but this education would be regulated by ONE FACULTY, and moulded into some kind of uniformity.

Does our contemporary imagine that, because in the extensive departments of France it is proposed to have several faculties, the education will differ in each of those, as it does in each of our colleges here? And what says our contemporary to the separation of pharmacy from physic there, and the establishment of one uniform system of education, where every practitioner must have his doctor's degree, before he can enter on the duties of his profession? What a terrible picture this for the domineering Scotch doctor to contemplate! What! a general practitioner to possess a degree—and to prescribe—and to throw pharmacy overboard! "The affair is *rank*—it smells to Heaven."

To the augmented scale of education, we would suggest to the legislature the utility of fixing the commencement of medical practice at a later epoch of life—say the age of 25 years—than at present. If a better classical and mathematical education be insisted on, in addition to more extended medical study, the age of 25 years will not be too late. We confidently prognosticate that, if a *proper* parliamentary inquiry be instituted, the apprenticeship system will be modified, and the scale of education greatly equalized, whatever may be the ranks and distinctions retained. Equalization of education is all we contend for:—the *real rank* of individuals will then soon find its level in the profession and in society at large. This is what the monopolists well know, and hence the cry against raising the general practitioner, in point of knowledge, to a level with the Scotch Doctor.

Finally, we implore the provincial towns to follow the example of the licentiates of London, and the Westminster Medical Society, to get up petitions to Parliament, praying for an inquiry by a Committee of the House of Commons. If they do not bestir themselves all will be lost, notwithstanding the confident prognostications of some of our contemporaries.

P. S. Our contemporary is in high glee at finding some select passages in one of the volumes of this Journal, which apparently clash with some parts of Dr. Johnson's speech in the Westminster Medical Society. There are few Reviews that have amounted to 20 or 30 volumes, in which clashing sentiments might not be pointed out—as the Edinburgh and Quarterly Reviews can testify. The Medical Gazette has very cleverly cut out certain passages from this Journal to serve its purpose. We shall indulge our contemporary with a few other passages from the same work, to shew that the Journal is not so much against its Editor as the Gazette imagines.

The paper from which the Gazette quotes was not written by the Editor, and for the following reason, which must carry conviction to every unbiassed mind. In the very same volume (Vol. VI. pp. 569) of the Journal Dr. Johnson has published a long paper on Medical Education, not anonymously, but *avowedly as his own*, in which he maintains all the opinions contained in his late speech at the Westminster Medical Society.

No. I.

"Mr. Lawrence's introductory lecture to his Spring Course has been published in the Lancet, No. 181, and the lecturer has exerted all his talents and rhetoric to prove, that no real distinction or line of demarcation can be drawn between physic and surgery, whether we look to education or practice. We shall here bring forward a document, shewing how far Mr. Lawrence has been original in his arguments and illustrations, on this point of medical jurisprudence. The following paper was written and published by the Editor of this Journal *ten years ago*, and may be seen in the 4th volume of the Medico-Chirurgical Journal, pages 342—7. It will be evident that Mr. Lawrence has taken the arguments, the illustrations, and almost the identical expressions of this paper for his introductory lecture.

"The boundaries between physic and surgery have not till this moment been defined—a strong presumption that nature does not sanction them! The distinction of internal and external diseases is any thing but correct or satisfactory. Gout, for instance, alternates with erysipelas. In the first form the physician claims it as his own; in the second, the surgeon steps in and demands the treatment. But is not the erysipelas in this case, and indeed in almost every other case, dependent upon an internal, or what is termed a constitutional cause? and can it be, with any degree of safety, treated as a topical affection? Here, then, must not Physic and Surgery be united?"—569.

No. II.

"Let us glance at the great disorders of the three principal cavities of the body. Is idiopathic phrenitis more dangerous and difficult to treat than that from a fracture of the skull? Are idiopathic convulsions more terrible than those from a splinter of bone driven through the coverings of the encephalon? Is idiopathic tetanus more fatal than traumatic? In what consists the difference between idiopathic and traumatic pleuritis, pulmonitis, carditis, diaphragmitis, hepatitis, gastritis, splenitis, enteritis, peritonitis, cystitis?—None. Or, if there be any difference, the difficulty and the danger are on the side of surgery.

What then remains? Fever? Does the hectic of the lungs exhibit a greater spectacle of horror and emaciation than the hectic of lumbar abscess, or disease of the hip-joint? Is the inflammatory fever resulting from aërial vicissitudes to be compared with that supervening on a lacerated member or crashed knee-joint, where the dire commotion threatens every instant to annihilate the living fabric at a blow? The typhoid fevers differ in no respect from the ulterior stages of inflammatory and symptomatic fevers.

Seeing, then, that the greatest and most dangerous diseases, both in physic and surgery, to which humanity is subject, are completely identified, is it not evident that the surgeon, in acquiring a knowledge of *these*, must, in course, acquire a knowledge of the minor and intermediate maladies, which are reckoned *Medico-Chirurgical* by both professions? And, is it not evident from these premises, that the surgeon, if properly qualified, must add to the *science* of the physician the *art* of operating? Anatomy, physiology, and surgery, are merely the first steps by which the student gains a footing on the hill of science. When he can unravel the brain without a light, or take up the external iliac blindfold, he is scarcely advanced a day's march on the great journey of medical science. These, which are termed, and justly, elementary branches, are little more than the alphabet of medicine, though they are considered at the beginning as the ultima thulé of professional knowledge. The surgical student thinks himself armed at all points; but every year's subsequent experience shews him more and more his own ignorance!

In fine, physic and surgery are only parts of a grand whole; one cannot be properly known without the other, no more than navigation can be learnt without the aid of arithmetic. The distinctions between the two branches cannot be drawn or maintained, even in the largest cities. To be convinced of this, let any one sit down by the side of Cooper and Abernethy for a day, and he will see these illustrious surgeons lopping off nine surgical cases with the quicksilver pill and infusion of cascarilla, for every one which they remove by the knife or by caustic. But what is more extraordinary, he will see patients of all ranks afflicted with purely *physical* diseases, resort to surgeons for advice. Hence it is evident, that the general sense of mankind acknowledges not the artificial divisions which we have made in the profession."

No. III.

"In no possible way can we find any natural or just distinctions between physic and surgery; and yet I do not object to the distinctions in *practice*. It is against the idea of a difference in *education and study* that the above remarks are levelled. It is morally impossible that a man can be a good physician without knowing the principles of surgery; and it is still more absurd, to think that a man can be a good surgeon without being intimately acquainted with the principles of physic. If it be objected that an intimate acquaintance with the minutiæ of both branches is too much for one person to acquire, I answer, 1st, in the words of Richerand, 'l'etendue de la sciénce ne justifie point les *limites arbitraires* que l'on voulut tracer entre ses diverses parties.' 2d. I am of opinion, that it is much more easy to acquire a profound knowledge of both branches, than of either one separately: and for the reasons already stated, that the two sciences are interlocked at all points, even the apparently most distant; for instance, *pneumonia and fracture*. Hence to attempt to learn the one without the other is to tie up one of our arms when we commence a mechanical trade!" *Med. Chir. Journ. vol.* IV. *p.* 344, 1817, *and Med.-Chir. Rev. vol.* VI.—1827.*

No. IV.

"But, except the purely *operative* part of surgery, medical and surgical *practice* is completely blended, for the best of all reasons, that medical and surgical diseases are essentially the same. Hence, it is incumbent on the young physician to *study* surgery at least, and to dissect as assiduously as though he were designed for the operation room; while the surgical student may be assured that *medicine* is his sheet anchor at last; and that whatever degree of accuracy he may attain as an anatomist, or dexterity as a surgeon, yet, without pathology and therapeutics, these brilliant acquisitions will avail him little in his professional journey through life." *Ib.*

We now come still closer to our present epoch—namely, to 1828.

No. V.

"We maintain, then, in the first place, that the PRACTICE of MEDICINE necessarily includes a knowledge of surgery—secondly, that the PRACTICE of SURGERY necessary includes the practice as well as the knowledge of medicine—and thirdly, that the PRACTICE of these two great artificial divisions,

---

* The Gazette cannot pretend that it only sets the Med.-Chir. Review against its Editor—his final couplet shews, that his object is to prove that Dr. J. is inconsistent with himself:—

"Manners with fortunes, tempers *change* with climes,

Tenets with books, and *principles with times*."

GAZ.

necessarily includes a knowledge of all the other divisions, however designated as subordinate, auxiliary, or collateral. Midwifery, pharmacy, chemistry, medical botany, ophthalmic, acoustic, and dental surgery, &c. are all as arbitrary dislocations as those of the unlettered butcher, the erudite Bonetus, or the experienced Baillie. To these separate branches let men attach themselves, (after being grounded by education in all the others,) as interest, inclination, or individual genius, may prompt. But, for our parts, we shall make no distinction between the different members of the same family."—*Med.-Chir. Review, Vol.* VIII. *Jan.* 1828.

With the exception of pharmacy, these are still our sentiments. The education should, and will be equalized, where the profession is one. A minimum raised to the height of the present maximum does not preclude men from going higher, nor withdraw the stimulus to industry. On the contrary, it would add to the stimulus.

*Note.*—It may surprise our readers that these "*levelling sentiments*" of Dr. Johnson, contained in the foregoing extracts, have never before attracted the attention of our aristocratic contemporary! There are reasons for all things. While the Medico-Chirurgical Review was in open hostility with the Lancet, Dr. Johnson was a respected fellow labourer in the eyes of the Gazette, but from the moment when he (wisely) determined to desist from all hostilities with the Lancet or other journals, the MEDICAL GAZETTE took every opportunity of censuring and carping at him! Dr. Johnson never once retaliated, and does not mean, even under all these galling annoyances from an old friend, to renew a system of hostilities, which do no good to either party, and injure the profession generally. The Gazette may act on this hint, and fire away, under the certainty that no shot will be returned.

### WESTMINSTER MEDICAL SOCIETY.

Our contemporary is nearly ready to jump out of his skin with joy, because, by a conservative manœuvre on one night, and a clique of monopolists on another, the motion for a faculty to regulate the education and the practice of the profession was negatived, after being carried by a great majority! The manœuvre was to mystify the business, and lead the Society to believe that the BALLOT was to be upon the *amendment* of Dr. Johnson's motion. By this manœuvre, the reformers were thrown off their guard—a conservative clique was brought down the next night—and the *original* motion was negatived by a paltry majority of *ten*. Even this insignificant majority would not have taken place in a jury, packed as it was, had not the MEDICAL GAZETTE distorted and misrepresented the intention of Dr. Johnson's motion. That motion made no allusion to the annihilation of colleges, or other seminaries of education now existing; but only to the establishment of a FACULTY, which should regulate the education of the whole profession, and prevent one college from issuing one curriculum of education, and a neighbouring college a totally different one; by which discrepancies and inconsistencies, great detriment was done to medical society and the public at large. Thus, for example, such a faculty would ordain that the colleges of London, Edinburgh, Glasgow, Dublin, Aberdeen, and St. Andrews, whether medical or surgical, should prescribe one uniform curriculum of study, leaving it to the students to graduate at any one of these colleges according to their convenience. This reasonable and useful proposition was instantly converted by the MEDICAL GAZETTE, into a proposal to abolish all existing institutions, and have but one MEDICAL DEMOCRACY in the three kingdoms! A more disingenuous and false interpretation was never put upon words!

But the MEDICAL GAZETTE may possibly have exulted somewhat prematurely. Dr. Epps has given notice for a discussion on the legality of the proceedings on the above occasion, which discussion will take place on the first meeting after Christmas. If the reformers sleep supinely on their posts, and permit the monopolists to carry their measures a second time, why then the cause of reform is not zealously entertained by those members. We want nothing but an open arena and fair play. Let the sense of the Society decide. The magnates of monopoly are now openly boasting every where, that Dr. Gregory has outwitted and knocked up the reformers—by first coinciding apparently with—nay, actually *penning*, the resolution which he afterwards opposed, and caused to be thrown overboard in the Society—thus launching the apple of discord among its members. Yes! Dr. Gregory undertook to draw up a resolution, from two or three propositions, that might meet the wishes and concentrate the ideas of all present —and this resolution, which Dr. Johnson undertook to move, was afterwards opposed and cancelled by Dr. Gregory in the general meeting!!! We envy him not his triumph—which may, perhaps, be short.

Should the original resolution be again agitated, Dr. Johnson means to word it in such a manner, that it cannot be misrepresented or misunderstood —and should it not be agitated, he will give notice of a motion to the following effect:—

"That, in the opinion of this Society, a more extended and more uniform system of medical education than at present exists is highly desirable; and that the power of organizing and regulating that system of education would be better vested in one body or faculty, under the sanction of the Legislature, than left to the option or caprice of various universities, colleges, corporations, and faculties, each of which enjoins its own peculiar course of study, whereby much confusion and discordance are produced in a profession, where the mode of elementary education ought to be the same for all its members."

If the monopolists of high-pressure education and academic honours come down to stifle this attempt to raise the general level of medical knowledge throughout all ranks of the profession —let them have the credit of their disinterested labours to themselves. We trust, however, that they will be disappointed.

